# Precision periodontal care: from omics discoveries to chairside diagnostics

**DOI:** 10.1007/s00784-023-04878-7

**Published:** 2023-02-01

**Authors:** Nagihan Bostanci, Georgios N. Belibasakis

**Affiliations:** Section of Oral Health and Periodontology, Division of Oral Diseases, Department of Dental Medicine, Karolinska Institutet, Alfred Nobels alle 8, 141 52 Huddinge Stockholm, Sweden

**Keywords:** Personalized dentistry, Precision dentistry, Periodontal diseases, Saliva, Gingival crevicular fluid, Chair-side diagnostics, Proteomics, Oral microbiology, Oral immunology

## Abstract

The interface of molecular science and technology is guiding the transformation of personalized to precision healthcare. The application of proteomics, genomics, transcriptomics, and metabolomics is shaping the suitability of biomarkers for disease. Prior validation of such biomarkers in large and diverse patient cohorts helps verify their clinical usability. Incorporation of molecular discoveries into routine clinical practice relies on the development of customized assays and devices that enable the rapid delivery of analytical data to the clinician, while the patient is still in session. The present perspective review addresses this topic under the prism of precision periodontal care. Selected promising research attempts to innovate technological platforms for oral diagnostics are brought forward. Focus is placed on (a) the suitability of saliva as a conveniently sampled biological specimen for assessing periodontal health, (b) proteomics as a high-throughput approach for periodontal disease biomarker identification, and (c) chairside molecular diagnostic assays as a technological funnel for transitioning from the laboratory benchtop to the clinical point-of-care.

## Etiological justification of oral diseases

Periodontal diseases are highly prevalent in the human population. Whereas we know a lot about the microbial and host afferents involved in their etiopathogenesis, we are not as efficient in applying this knowledge into the daily clinical practice for the benefit of the patients. To justify this mismatch between experimental knowledge and clinical application, one should consider some fundamental differences between oral and medical infections. Traditional medical infections are preceded by the colonization of the affected site by an external pathogen, which will invariably invade a tissue and lead to the development of infection within. In this instance, detection of single pathogen has diagnostic value, whereas targeted elimination of pathogen is a meaningful treatment outcome. Nevertheless, in oral infectious disease, there are no sole pathogens that stand out as etiological agents [1]. Shifts in oral microbial communities can lead to a disadvantageous relationship between the host and its resident microbiota, a phenomenon which is best described as “dysbiosis” and may eventually locally compromise oral health. We have therefore adopted an “ecological” consensus on the etiology of oral polymicrobial diseases, such as and periodontitis [2]. Hence, singling out the detection of a sole microorganism among the polymicrobial plethora cannot fulfill a diagnostic role for periodontitis.

## Technological advancements and methodological limitations of omics

Technological advancements in molecular sequencing shed light to the “microbial dark matter” obtailed from clinical sites of infection. This is the part of the microbiome that was earlier not “visible” to researchers by conventional cultivation methods. Sequencing technologies have confirmed the presence of already known taxa but have also identified dozens of other unknown or unsuspected taxa in a given sample [3, 4]. An important realization is that the members of these taxonomically diverse microbial communities display overlapping functional and metabolic activities in disease [5, 6]. It is therefore important to identify microbial or molecular “trends” that occur during conversion from health to different stages of the disease rather than just individual bacteria or molecules [7]. The information gathered by omics technologies has so far been used to extensively catalogue the microbes or proteins, clustering them into “core” microbiomes or proteomes, according to the clinical status of the patient or the affected site. Nevertheless, extensive optimization of microbiome-based clinical studies needs to be achieved for ensuring compatibility and comparability across studies [8]. Many technical confounding factors need to be resolved, such as adhering to minimum quality standards in sample collection and storage, data reporting and deposition, development of clinical user-friendly software tools, and validation of the findings in several populations.

## Conceptualization of personalized oral healthcare

**Fig. 1:**
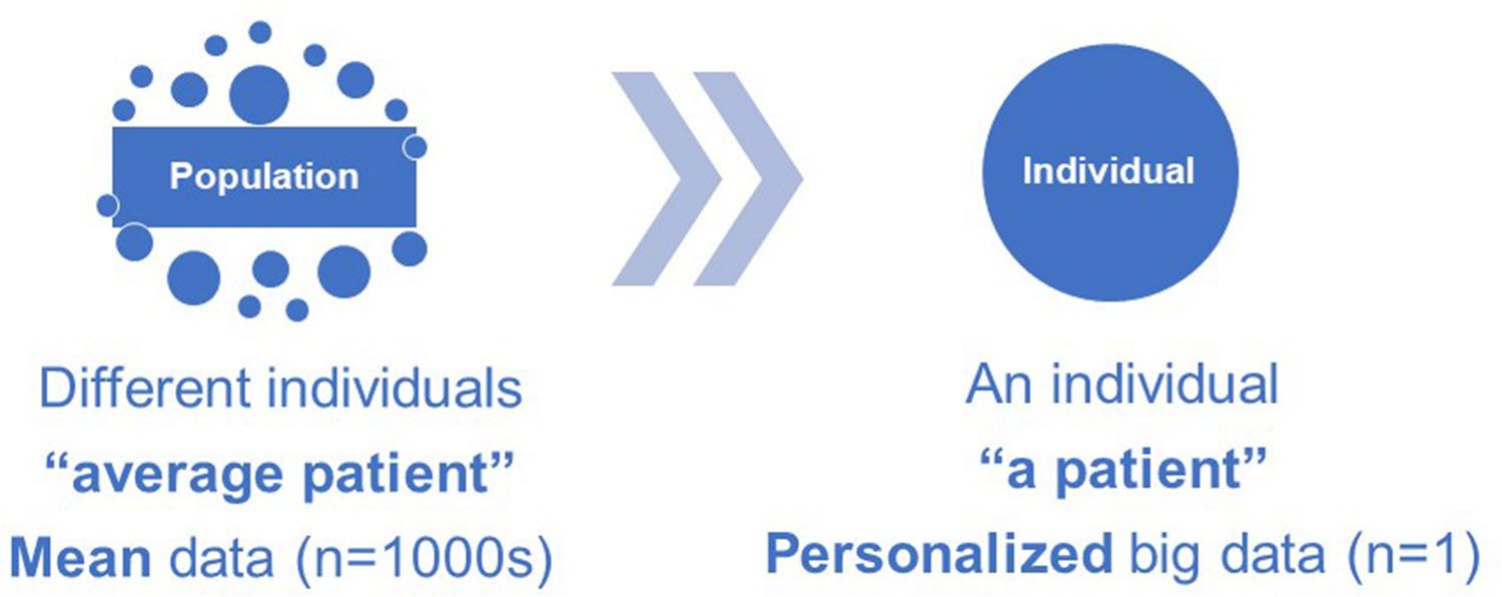
Personalized healthcare is the tailored delivery of care to an individual patient, away from population-based conclusions. In terms of diagnostics, rather than deducing an average single biological measurement from the whole population and applying it on an individual patient, multiple biological measurements are registered on a single individual, before reaching a medical conclusion

Dentists and physicians have long recognized symptomatic variations among patients and have strived to customize the delivery of care based on each individual patient’s health needs. Personalized medicine traditionally refers to the individualized healthcare care based on a person’s unique clinical characteristics, environmental exposures, and behavioral traits [9, 10]. In the case of periodontal diseases, personalized emphasis is placed on addressing risk factors shared with other health conditions, such smoking and cardiometabolic diseases, which is a reasonable and efficient approach for improving oral health in large segments the population [11]. Yet, adjustment of population-level behavioral risk factors is not utterly precise for guiding personalized periodontal healthcare [12]. We know from studies on the natural history of periodontitis that the escalation pattern of the disease varies among patients [13], as does the predictive response to treatment [14–16]. While clinical observations clearly indicate that variations in oral disease patterns are attributable to heritable factors [17], specific genomic evidence to pin-point common non-syndromic forms of periodontitis is still inconclusive [12]. Genome-wide polymorphisms can only modestly explain variations in the clinical presentation among patients, whereas current diagnostic processes and classification systems are based on the registered clinical symptoms and signs, without integrating any discovery-driven genomic, epigenomic, or proteomic information [18, 19]. As such, we tend to extrapolate conclusions or formulate guidelines based on the “average patient” and then apply the “average treatment” to a single individual [20]. Nevertheless, personalized healthcare requires tailoring diagnosis and treatment to a single patient, after screening not only for individual clinical measures, but for also multiple behavioral, environmental, and biological determinants of health (Fig. 1).

## Current state of personalized oral healthcare

Personalized healthcare is being redefined as precision healthcare, meaning that molecular diagnostic aids that disclose an individual’s unique genetic and biological profile may help deliver a more focused diagnosis and treatment [21, 22]. Studying complex biological systems on a single patient though entails a holistic approach that incorporates a wide variety of disciplines to collectively personalize the patient’s “big data” (Table 1), while prospecively monitoring any occuring changes (Table 2). The currently available diagnostic armament of the clinician includes primarily the clinical and radiographic examination, whereas auxiliary histological, microbiological, or biochemical determinations can be requested on occasion. These are usually performed at specialized referral hospital-based laboratories.

**Table 1:** Considerations for personalization of big data

Biological data resource: microbiome, proteome, metabolome	
Measurable traits: differentiation between transient physiological and persistent pathological changes	
Read out: development of reliable analytical tools with well-defined cut-off diagnostic criteria	
Feasibility: rapid test assays and applicable at point-of-care (POC)

**Table 2:** Strategies of monitoring the oral microbiome

Sampling approach:	
▪ Site-specific: dental plaque or gingival crevicular fluid	
▪ Region-specific: saliva or buccal swabs	
▪ Systemic: blood	
Monitoring traits:	
▪ Microbial community composition: metagenomics, proteomics	
▪ Community metabolic functions: meta-transcriptomics, proteomics, metabolomics	
▪ Surrogate markers: selected “core species” or “signatures” of species	

With regard to periodontal diagnosis and therapy, some basic approaches to screen for biochemical or molecular factors in biological fluids, such as saliva or gingival crevicular fluid (GCF), have been implemented over time, reaching even the clinical market on a few occasions. As such, antibody-based host–enzyme detection assays for granulocyte-derived active matrix metalloproteinase (MMP)-8 in biological fluids can serve as good predictors of undiagnosed periodontitis, potentially with higher specificity than some of the clinical parameters [23–26]. Enzyme-based assays for bacterial protease activity in saliva or GCF may predict the presence of certain periodontal pathogens at the level of the affected site or patient [27, 28].

Physical methods, such as broad-spectrum fluorescence resonance energy transfer, can measure the total protease activities in saliva and may predict the long-term magnitude of the gingival inflammatory response of an individual [29]. Infrared attenuated total reflection (IR-ATR) spectroscopy has also been used to screen for inter-individual differences in saliva, demonstrating a good capacity to discriminate between non-periodontitis and periodontitis-affected individuals [30]. Secondary electrospray ionization (SESI) is shown to successfully identify individual metabolites of periodontal pathogens in saliva, commensurate with a clinical diagnosis of periodontitis [31]. Fluorescence-based methods used for monitoring dental plaque development on teeth are based on exploiting the plaque’s inherent capacity to emit red fluorescence [32]. The level of red fluorescence emitted from accumulated plaque as early as 24 h could predict the level of gingival inflammation over a few weeks later [33]. This property could support the stratification of patients according to low- and high-risk groups for developing gingival inflammation, potentially revealing their susceptibility to periodontal disease. The representative molecular, biochemical, or physical detection possibilities that were described in this section summarize the need to utilize a broad range of technological platforms and biological parameters (both microbial and host) in the diagnostic chairside armament for oral diseases.

## Salivary proteomics discovery for personalized periodontal healthcare

The advances in saliva brought “omics” sciences as a feasible approach to unravel molecular patterns of periodontal diseases [34, 35] and hence put precision periodontology closer to clinical practice. Saliva is a unique body fluid for on-site real-time monitoring, amenable to a wide range of possibilities for diagnostic applications. From the 1980s onwards, investigation of salivary proteins gained momentum with the understanding of whole saliva as a mixture of proteins pooled collectively from GCF, serum, and microbiota and of course the three pairs of major and the numerous minor glands. In systemically healthy individuals, the salivary protein composition and levels are relatively stable over longer periods of time [36] and may well reflect serum composition [37], whereas the fraction of proteins showing inter-individual variability makes salivary proteome a useful medium for biomarker identification [38]. The evolution from gel electrophoresis assays that enabled separation of proteins by size/charge, to the mass spectrometry sequencing platforms, able to sequence multiple proteins in a single run, has led to a giant quantitative leap in salivary protein discovery in saliva from less than 20 [39] to more 1000 in any given sample [40].

**Fig. 2:**
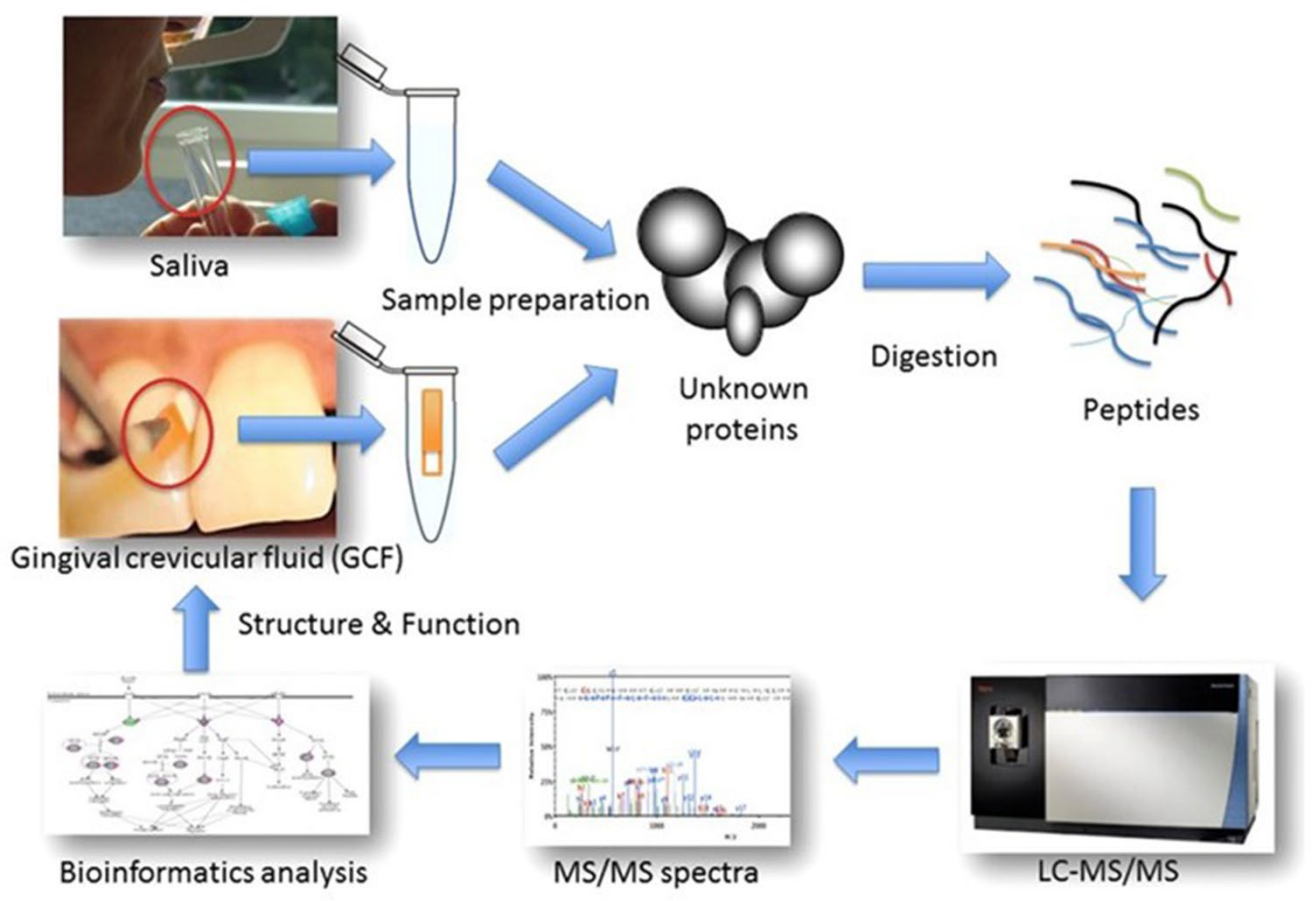
Workflow for shotgun proteome analysis of gingival crevicular fluid (GCF) and saliva. While GCF is collected over a period of 30 s by insertion of a filter paper into the gingival crevice or periodontal pocket, whole saliva samples are obtained simply by expectorating into polypropylene tubes. The obtained protein extracts are used for bottom-up shotgun proteomics. Reproduced from [34] with permission from Wiley

**Fig. 3:**
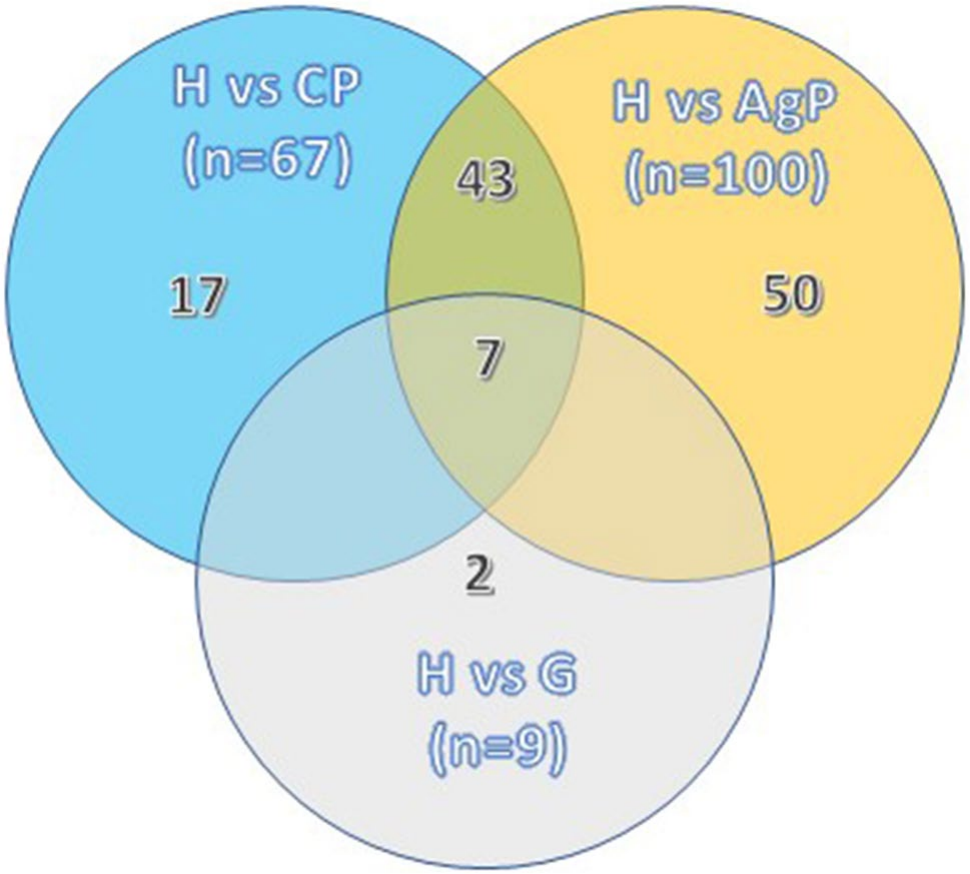
Venn diagram summarizing the number of differentially expressed proteins (*p* value <0.05 and ≥2-fold) and overlaps between health (H) and the diseased groups (G, AP, CP) by using label-free quantitative proteomics (LFQ). Gingivitis (G), aggressive periodontitis (AP), chronic periodontitis (CP). Adapted from Bostanci et al. Mol Cell Proteomics 2018 [34]

In general, proteomic applications can be divided into bottom-up and top-down strategies, where proteins are either digested into peptides or used in their entirety, respectively. The former approach is mainly used for GCF and salivary proteomics (Fig. 2). When coupled to mass spectrometry innovations or combined with “targeted” proteomics, they can deliver a more accurate analysis of the salivary proteome, with clinically relevant implications, such as differentiation between clinical health states. In one of our studies using this “two-step” pipeline, we were able to comprehensively map the salivary proteome and discover many less known regulated proteins than those previously established in the literature [41]. In the first methodological step of the process, we identified and quantified over close to 500 proteins in patients’ saliva, the vast majority of which were of human origin, but bacterial and fungal ones were also present. In the second methodological step, 60 of those proteins were additionally quantified, eventually pin-pointing a panel of 5 biomarkers with high predictive value for periodontal diseases. Those 5 biomarkers could be exploited future chairside diagnostic applications. From the totality of identified salivary proteins, approximately 100 were differentially abundant between health and periodontitis whereas less than 10 between health and gingivitis (Fig. 3). This narrow difference between health and gingivitis does not exclude the possibility that subclinical inflammation is present in clinically healthy individuals, which nevertheless cannot be detected by standard diagnostic means (i.e., clinical examination).

Experimental gingivitis models have indeed identified the differential susceptibility of individuals in developing gingival inflammation in response to dental plaque accumulation. They can be classified into “slow” responders, who display a delayed onset of inflammation, and “fast” responders who respond immediately to plaque accumulation [42]. Global proteomics or targeted antibody-based analyses of saliva in those individuals have indeed identified that the protein profiles of those two groups vary significantly [25, 43–46]. In the “fast” responders, almost 50 proteins were detected at >4-fold higher levels than the “slow” responders. Such differential trends in protein expression are defined as “proteotypes” that can prognosticate individual susceptibility to gingival inflammation. A “proteotype” is the dynamic state of a proteome at a particular time point [47], and thus, one would expect that any given proteome would have consisted of many proteotypes [48].

The combined application of proteomics, genomics, transcriptomics, epigenomics, and metabolomics technologies for salivary analysis is collectively defined as “salivaomics” [49]. Yet, it is currently more feasible to generate fragmented rather than combined omics datasets with these distinct approaches. Due to their methodological and bioinformatic complexity, multi-omics applications are still pending further clinical validation and verification for their usability in saliva or GCF.

## A paradigm of chairside salivary diagnostics at the point-of-care

**Fig. 4:**
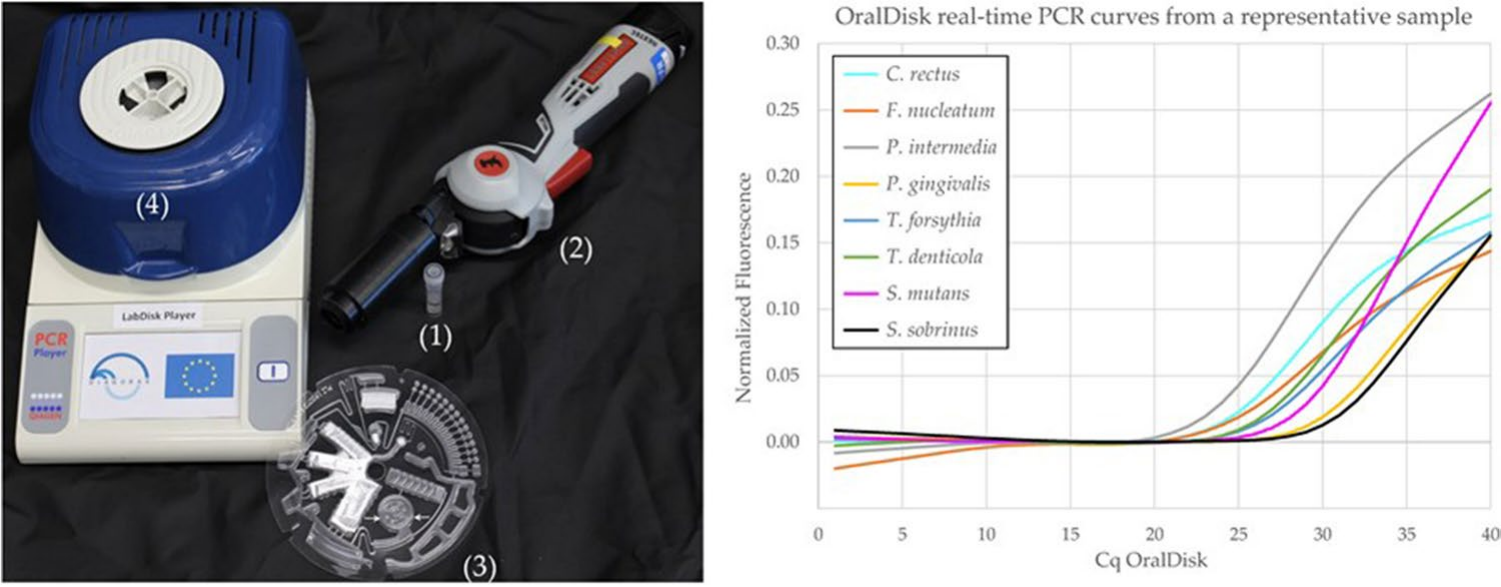
(Left) Items used in the process, with the tube containing the saliva sample and steel beads (1), a hand-held instrument used for performing mechanical lysis and homogenization of the salivary sample (2), the disk cartridge (OralDisk) on which the analysis takes place (salivary sample is pipetted in the chamber indicated by white arrows), and (3) the LabDisk Player instrument that performs the qPCR on the OralDisk (4). (Right) Representative qPCR curves for various bacteria detected by the OralDisk in a salivary sample. Published in Baumgartner et al. Biosensors (2021) [59] and reproduced according to the Creative Commons Attribution License permit

Personalized dentistry should ideally take place in any suitable point-of-care (POC) hub. This may include the general medical and dental practice offices, nursing homes, outpatient clinics, and potentially self-testing at home. This entails the use of technological devices on the patient’s chairside, with the capacity to detect in short time selected biomarkers within a given patient sample. Such an approach to bring personalized dentistry closer to clinical practice has been addressed by the “DIAGORAS” EU Horizon 2020 project (www.diagoras.eu), which amounted 5.5 million euros and occupied 10 academic and corporate partner organizations from 8 European countries. The project aspired to improve the chairside options for personalized monitoring and diagnosis of oral and respiratory tract infections [50]. The oral healthcare branch of the project specifically aimed at developing chairside microbiological and immunological assays, scaled down to a methodological utility at the size of a conventional compact disk. This “lab-on-disk” cartridge is readable on a custom-built reader device, suitable for easy use at diverse POC hubs. The disk has integrated packed chemical reagents for qPCR assays, whereas the reader is designed to perform all functions of a thermal cycler (Fig. 4A). The qPCR protocol was developed originally as a POC-compatible benchtop assay on a pre-defined panel of bacterial targets in saliva, including periodontal pathogens and cariogenic species [51]. The assay was thereafter successfully integrated into a disk cartridge (“OralDisk”) (Fig. 4B), which displayed 90% qualitative agreement in bacterial species detection, as compared to its parental benchtop assay [52]. Inventions such as the “OralDisk” are highly adaptable for various microbial targets. They can, for instance, be implemented in detecting antibiotic resistance genes in samples obtained from oral infections [53].

A series of studies on clinical patient cohorts validated the suitability of several bacterial and host biomarkers in saliva, by confirming their ability to distinguish between oral health and disease (e.g., dental caries, gingivitis, periodontitis) [51, 52, 54]. While not fully yet implemented in clinical practice, the product of the DIAGORAS project, the integration of host immune salivary biomarkers in chairside POC diagnostics also holds great promise. The development of fully automated pre-analytical salivary protein processing and the validation of one-step heterogeneous immunoassays for saliva could lead to fully automated rapid sample-to-answer utilities, such as the “ImmunoDisk” [55–57]. The advantage of the technological chairside molecular detection platform(s) described in this section is the rapid salivary sample analysis and the immediate delivery of microbiological or immunological data while the patient is still in session, when this information is most useful and usable to the clinician.

## Conclusions

Personalization of oral care for managing oral infectious diseases, such as periodontitis, is currently more foreseeable via the prism of diagnostics and prevention strategies. The concept has practically been here for long, but scientific and technological gaps have been limiting its routine application, whereas further adjustments are needed to validate this [10]. Proteomic discoveries have helped us pin-point possible molecular targets, or combinations thereof, that can be used as biomarkers in saliva and other biological fluids [58]. Coupling these biological discoveries to the development of cost-effective, non-invasive, rapid analytical technologies will enable the utilization of biomarkers at the chairside POC, solidifying the precision element of personalized oral healthcare.
